# Justifications for Judgment Accuracy in Sports

**DOI:** 10.3390/sports13040120

**Published:** 2025-04-14

**Authors:** Athanasia Chatzipanteli, Aglaia Zafeiroudi, Ioannis Trigonis, Ioannis Tsartsapakis, Alexandros Fotiadis, Asterios Patsiaouras, Nikolaos Digelidis

**Affiliations:** 1 Department of Physical Education and Sport Science, University of Thessaly, 42100 Trikala, Greece; azafeiroudi@uth.gr (A.Z.); alfotiadis@yahoo.com (A.F.); spats@uth.gr (A.P.); nikdig@uth.gr (N.D.); 2 Department of Physical Education and Sport Science, Democritus University of Thrace, 69100 Komotini, Greece; itrigon@phyed.duth.gr; 3 Department of Physical Education and Sport Science, Aristotle University of Thessaloniki, 62122 Serres, Greece; ioantsar@phed-sr.auth.gr

**Keywords:** judgment accuracy, actual performance, volleyball serve, procedural knowledge

## Abstract

This study investigated the causes of incorrect judgments in a motor task and examined differences between students with varying levels of judgment accuracy. Twenty-two seventh graders participated. Based on their estimated and actual scores in two volleyball serve trials, students were categorized into two groups: “low accuracy” and “high accuracy”. Before each trial, they estimated their scores according to the American Alliance for Health, Physical Education, Recreation, and Dance test. Following the trials, students were interviewed about their justifications and their confidence in the accuracy of their judgments. Independent sample *t*-tests indicated that both “low accuracy” and “high accuracy” students appeared to use metacognitive skills (*t*(20) = 0.82, *p* > 0.05). However, the “low accuracy” group lacked the declarative and procedural knowledge (*t*(20) = 4.59, *p* < 0.001) necessary for accurately evaluating their performance. Findings suggest that students focused more on outcome-based rather than process-based assessments when evaluating their performance. Enhancing students’ access to both theoretical and experience-based cues in sports may improve their ability to accurately judge their performances and foster greater confidence in lifelong participation in physical activities.

## 1. Introduction

Metacognitive judgments provide insight into how well a learner has understood or mastered a task [1]. In educational settings, it is preferable for students to accurately assess their own knowledge, as more precise monitoring leads to better self-regulation, effective control, and improved performance outcomes [2]. When learning a new skill, students who accurately evaluate their own progress can focus better, adjust their study efforts, or even modify their learning strategies, et al. [3,4]. In contrast, inaccurate judgments can result in inefficient decision-making and poor learning outcomes [3].

The accuracy of judgment depends on several factors. According to the Kruger–Dunning effect, overestimation occurs when students lack metacognitive skills, such as monitoring and reflection, preventing them from recognizing their poor performance [5]. Ortega et al. [6] highlight that judgment performance is influenced by both theory-based and experience-based cues. Theory-based cues involve prior knowledge within a particular domain, whereas experience-based cues relate to the procedural fluency of performing a specific task [7]. The inappropriate application of either type of cue can lead to inaccurate judgments.

High-performing learners seem to use theory-based elements as they possess greater knowledge, their understanding is more structured, and their information processing is more efficient [8]. They demonstrate greater domain-specific knowledge, which enables them to establish more connections among concepts, enhancing their problem-solving abilities that, in turn, influence experience-based elements [9]. For instance, a study found that elite divers verbalized more detailed concepts, such as arm and leg positioning and body alignment, and employed a greater variety of strategies (e.g., visualization, self-talk, etc.) compared to novice divers [10]. In contrast, poor performers often focus on irrelevant information and/or tend to use general skill-execution concepts [11]. However, general skill-execution elements such as concentration and focus are sometimes ineffective, as performers may not know how to focus or lack the necessary knowledge to direct their attention appropriately [12].

Regarding judgment accuracy predictions, low performers or non-elite athletes tend to overestimate their performance and exhibit overconfidence in their abilities. This phenomenon, known as the “skilled and unaware effect” [9], occurs when unskilled learners lack knowledge of the content being assessed and fail to recognize that they are inadequately prepared. However, evidence suggests that low performers do engage in monitoring processes, but other factors seem to contribute to their inaccurate judgments [10]. As a result, while students predict that they will perform better in the future, their confidence in the accuracy of their judgments remains low [11].

The finding that overestimation among low performers is associated with low confidence indicates that some low-performing learners may be aware of the inaccuracy in their judgments [11]. According to Saenz et al. [12], low-performing students’ judgments may be based more on their expectations than on their actual achievements. Research also shows that, despite opportunities for students to base their judgments on prior experiences, desired performance persists even in future task performance [10,13]. This suggests that wishful thinking influences judgment accuracy.

Additionally, some researchers believe that students may hesitate to admit that their performance is below socially accepted standards [12]. The fact is that although low performers aspire to improve, they do not necessarily aim for the highest possible score. Instead, they express their performance expectations based on their own abilities and prior achievements [10].

Numerous studies have examined the accuracy of learners’ judgments in the cognitive domain, but limited research has focused on the psychomotor domain, although students’ performance is also influenced by theory and experience-based characteristics. More specifically, no study has investigated how students perceive their abilities when performing motor skills in physical education (PE) classes.

Physical education plays a crucial role in developing motor skills and knowledge that enable individuals to engage in lifelong physical activities [14]. Accurate self-assessment of abilities can enhance performance outcomes.

Thus, the aim of this study was to investigate (a) the factors influencing students’ judgment predictions (e.g., metacognitive strategies, wishful thinking), (b) whether or not students are aware of the accuracy of their estimations, and (c) if there are any gender differences in judgment accuracy. It was hypothesized that: (a) Factors such as metacognitive strategies and wishful thinking are positively related to students’ judgment predictions, and (b) students are aware of the accuracy of their estimations.

Regarding the third research question, no specific hypothesis was stated. We could not hypothesize if there was any difference between boys and girls in their volleyball serve judgment accuracy.

## 2. Materials and Methods

### 2.1. Participants

The study included 22 seventh-grade students (13 boys and nine girls). All students had completed an eight-lesson volleyball unit and had also received volleyball instruction for eight lessons in both fifth and sixth grade. None of the participants were involved in volleyball clubs outside of school. The sample size used in this research was according to the recommendation that the minimum sample for a qualitative interview study could be between 20 and 30 individuals [15,16]. Particularly, when the study’s goal is to investigate the perceptions and experiences of relatively homogeneous individuals, 12 interviewees might be sufficient [17]. Ethical approval for the study was granted by the University Ethics Review Committee. Participation was voluntary, and informed consent was obtained from the students’ parents prior to their involvement in the study.

### 2.2. Measures

**Figure 1:**
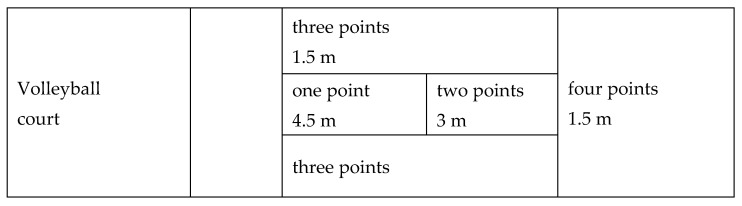
American Alliance for Health, Physical Education, Recreation and Dance motor skills test for volleyball service. m = meters.

The American Alliance for Health, Physical Education, Recreation, and Dance (AAHPERD) [18] (Figure 1) motor skills test for the volleyball serve was used to assess students’ performance. The test has acceptable validity and reliability (80%) [19].

According to the AAHPERD test, students were required to execute serves on a volleyball court. For valid serves, they were awarded points ranging from 1 to 4, depending on the marked area where the ball landed on the opposite court. Specifically, if the ball landed near the baseline, the student received four points. If it landed on the left or right sides of the court, they received three points. Serves landing in the area between the net and the baseline earned two points, while those landing near the net were awarded one point. Invalid serves received a score of zero, indicating a failed attempt.

As both boys and girls practiced during physical education courses, the volleyball court in our study measured 9 by 18 m, with a net height of 2.24 m.

### 2.3. Procedure

The volleyball unit was taught over eight weeks in accordance with the school curriculum. During this period, students learned and practiced key volleyball skills, including the overhand pass, forearm pass, underhand pass, and overhand serve. Upon completing the unit, they were asked to estimate their volleyball serve scores based on the AAHPERD test’s scoring structure. Subsequently, they were required to recall key aspects of an effective serve, such as technical execution, appropriate force, body posture, and follow-through, to direct the ball to a specific area. Before their first attempt, students were asked: “How many points do you believe you will score when you perform the volleyball serve?”.

The researchers recorded the estimated scores, after which students performed their first volleyball serve (first trial). This required students to predict their score before immediately executing the serve. The points awarded for their performance represented their actual score. After completing the first trial, students repeated the same procedure for a second trial, involving another round of estimation and execution. Following both trials, students were interviewed about the strategies they applied and their level of confidence in their performance.

During the students’ volleyball performance, the two researchers recorded the actual scores based on the AAHPERD test. Inter-rater agreement was calculated, and Cohen’s kappa (κ = 0.95) indicated high overall reliability. According to the two common measures of judgment accuracy—absolute accuracy and bias/estimation [20]—students were categorized into two groups: “low accuracy” and “high accuracy”, based on the difference between their estimated and actual performance scores. Students who consistently overestimated their scores by two to three points in both trials (bias) were classified as having “low accuracy”. In contrast, “high accuracy” students were those who had at least one accurate judgment (absolute accuracy) and estimated the other trial within one point of their actual performance.

### 2.4. Interview

A semi-structured interview was conducted to explore participants’ perspectives on their use of metacognitive strategies and their confidence in their abilities. Each interview lasted approximately 30 to 40 min. In line with theory- and experience-based cues, students were asked to explain how they justified their performance estimations by responding to open-ended questions (Table 1). These questions were designed to gain insight into how students form judgments about their own learning [7,21].

### 2.5. Justifications for Judgments

Students’ responses to the open-ended questions were classified according to theory-based and experience-based elements into the following categories: monitoring/reflection, declarative/procedural knowledge, experience, and confidence (Table 1). All interviews were recorded and transcribed verbatim, and students’ responses were coded by two independent coders according to these categories. Kappa analysis revealed a high inter-coder agreement (95%).

More specifically, students’ responses indicating confidence in their scores were coded as “1”, while those expressing low confidence or uncertainty were coded as “0”. Students who engaged in monitoring or reflection on their first performance received a “1”, whereas those who did not employ metacognitive skills were assigned a “0”. For the declarative/procedural knowledge category, students who demonstrated the use of specific and general strategies during their performance were given a “1”, while those who relied only on general strategies received a “0”. Similarly, students who considered their past experience in making judgments were coded as “1”, whereas those who did not refer to their prior experience were assigned a “0”. Finally, students who felt confident about their actual performance were coded as “1”; those with low confidence were assigned as “0”.

The interview questions and corresponding classification categories are presented in Table 1.

### 2.6. Data Analysis

First, descriptive statistics (frequencies) were calculated for students to be categorized in the “high” and “low accuracy” groups. Classification was conducted in order to find out how “high” and “low accuracy” groups view their performances and whether there are any disparities between the two groups’ perspectives. Furthermore, boys and girls were categorized into “high” and “low accuracy” categories, using chi-square analysis. Then, separate independent sample *t*-tests were calculated first to check potential differences in judgment prediction variables, including monitoring/reflection, declarative/procedural knowledge, experience, and confidence and following to investigate variations in the same parameters by gender. IBM SPSS Statistics version 26 was used to analyze all of the data, with the *p*-value set at 0.05.

## 3. Results

Firstly, it is important to note that most students slightly overestimated their performances. Specifically, 13 students overestimated their performances in both trials and were categorized as the “low accuracy” group, whereas nine students accurately predicted their performance in at least one trial and were classified as the “high accuracy” group (Table 2).

Frequencies and percentages for “high” and “low” judgment accuracy students are presented in Table 2.

Regarding the results from the chi-square analysis where boys and girls categorized in “high” and “low” accuracy groups are presented in Table 3.

Regarding the first research question, students appeared to utilize metacognitive skills. All participants employed metacognitive strategies such as monitoring, reflection, and replanning (Table 3). For instance, students stated:

*“I tried to correct my mistakes that I noticed in the first trial* (monitoring and reflection)* […] in the second trial, I tried to be more concentrated and changed my body posture”* (planning).

*(Student 1)*

*“In the second trial, I tried to change the technical elements of the serve, * i.e.*, my posture, and look at the court to focus on my goal* (monitoring and planning), *but I could not achieve the score I predicted”.*

*(Student 2)*

*“I felt confident even from the first trial […] and when I could not pass the ball over the net, I tried to hit the ball in a different way”* (monitoring, reflection, and replanning).

*(Student 4)*

*“I did not feel ashamed about the zero in my first trial; I tried to fix my mistakes in the second trial, so I changed how I hit the ball”* (monitoring, reflection, and replanning).

*(Student 7)*

*“I was very focused in the first trial; I saw how I served the ball, and I adjusted the force and the posture of my legs*” (monitoring, reflection, and replanning). *“I was very confident about myself in the second trial, too”.*

*(Student 10)*

On the other hand, findings revealed that “low accuracy” students primarily focused on general knowledge aspects such as concentration and force to improve their performance in the second trial. In contrast, “high accuracy” students incorporated both general knowledge (e.g., concentration, attention) and specific knowledge (e.g., technical elements of the motor skill) (declarative/procedural knowledge) (Table 3).

In response to the second research question—whether students were unaware of their estimations—students from both groups appeared confident in their judgments across both trials (Table 3). However, students in the low accuracy group, despite overestimating their abilities, were not confident in how close their estimations were to their actual performance. They expressed hope that they would improve in the next or future trial. For instance, they stated,

*“I hoped to achieve the score I predicted; I was sure about myself and that I would succeed”.*

*(Student 15)*

*“I did not feel confident about myself or my prediction. Maybe if I had practiced more in volleyball. Anyway, I hoped that I could do better”.*

*(Student 20)*

*“I wanted so much to succeed, so I was not disappointed by the first 0 score. I tried to do my best in the second trial […] in the second trial, I felt very confident but again, I failed to score”.*

*(Student 17)*

Other students also believed they would improve after correcting their mistakes from the first trial but ultimately failed to achieve their predicted scores. Some stated they did not clearly remember how to execute the skill (declarative knowledge), while others admitted they had not practiced the volleyball serve enough (procedural knowledge). For example, some commented:

*“If there were a zero-point option in the test, I would have chosen it for my performance estimation* *”.*

*(Student 8)*

*“I did not remember very well how to execute the serve, but when I saw that I passed the ball over the net to the court, I was sure I could do better next time”* (indicating doubt about her declarative knowledge).

*(Student 11)*

Regarding the effect of the first trial experience, most students appeared to consider their actual scores from the first trial (Table 3). Notably, although half of the “low accuracy” students continued to overestimate their abilities, they neither claimed nor expected to achieve the highest possible score. Instead, they anticipated gaining between one and two points.

*t*-test values, and descriptive statistics (mean and standard deviations) for the examined variables are presented in Table 4.

Regarding the third research question, data analysis showed significant differences between boys and girls in all judgment accuracy variables. Girls seemed to feel less confident and to employ limited metacognitive skills such as monitoring and declarative/ procedural knowledge.

*t*-test values, and descriptive statistics (mean and standard deviations) for gender judgment accuracy variables, are presented in Table 5.

## 4. Discussion

The present study investigated (a) the factors influencing students’ judgment predictions, (b) whether students were aware of their estimations in a motor skill task, and (c) if there are any gender differences. The findings suggest that while students employed metacognitive skills, their ability to accurately judge their performance was hindered by a lack of declarative and procedural knowledge. These results provide insights into the theory and experience-based elements that contribute to judgment accuracy in physical education classes.

Regarding the first research question, most students seemed to monitor their performances to reflect and re-plan their second execution, although they did not accurately judge themselves, maybe due to the lack of specific skill-execution elements. Students reported that their first trial influenced how they approached their second attempt, indicating an awareness of their own performance. Our findings are not consistent with the notion that learners who cannot accurately estimate their abilities lack metacognitive skills (Kruger–Dunning effect), maybe because sports tasks differ from other cognitive subjects. In sports, a product-oriented assessment is immediate feedback for learners that helps them to understand that they did not achieve their goal. As a result, they understand that they have to make changes. However, despite using metacognitive strategies, many students still misjudged their abilities, suggesting that their assessments were based on incomplete or inaccurate knowledge about the motor skill.

One key issue identified in the study is that low performers relied predominantly on outcome-oriented assessments rather than process-oriented evaluations of their performance. Instead of analyzing how well they executed the skill in terms of technique, they focused primarily on whether or not they succeeded in getting the ball over the net. This suggests that while they engaged in monitoring and reflection, their judgments were not always informed by a deep understanding of the movement mechanics required for successful execution. Similar findings have been reported in previous research, highlighting that students often prioritize external outcomes over internal movement processes when evaluating their performance [22].

Additionally, differences were observed between students classified as “low accuracy” and “high accuracy”. While both groups demonstrated metacognitive engagement, students with lower accuracy relied more on general strategies such as concentration and force, whereas high-accuracy students were more likely to incorporate specific technical adjustments (a well-located toss, the contact ball-hand, etc). Perhaps a lack of declarative/procedural knowledge could prevent low performers from seeing their own mistakes clearly, giving them the impression that they are performing much better [23]. This illusion could lead to “wishful thinking”, the hope of performing better in future trials, that may overshadow the experience of previous performances, increasing overconfidence.

Regarding the second question, whether or not students are unaware of their estimations/overestimations, the results showed that they continued to overestimate their performance, even though they were not accurate in their first prediction. The phenomenon of overestimation was prominent among students in the low-accuracy group. Many students continued to overestimate their performance, even after experiencing inaccuracies in their first trial. This finding aligns with the concept of wishful thinking, where students persist in believing they will improve in the future despite limited evidence supporting that expectation [10]. Their confidence appeared to stem from their expectation to perform better next time rather than from an objective evaluation of their skills.

Importantly, no students predicted the highest possible score, and many expressed uncertainties about their estimations. For example, they anticipated that their ball would land in the larger, simpler areas of the court, scoring one or two points instead of in the more challenging, smaller areas. Consequently, we can conclude that students are aware of their abilities/inabilities even when they overestimate themselves, maybe due to their desire for good scores. Our findings are in line with other studies demonstrating that low performers express their desires according to their own possibilities and/or their prior achievements [10].

Regarding the third question, the results showed that girls scored lower than boys in all variables, particularly in self-confidence, experience, and declarative knowledge. Our findings are consistent with previous studies that revealed females to be less confident than males [24]. It is possible that this occurs because experience improves motor skills and self-confidence. Young women are offered fewer opportunities in school settings since physical educators and coaches seem to be more focused on the boys’ programs [25]. On the other hand, in out-of-school settings, girls have limited access to safe and comfortable physical infrastructures and facilities, and many professional sports fields remain highly male-dominated [26].

Overall, the problem is that most students, particularly girls, seem to lack enough declarative and procedural knowledge, which in turn leads to inaccurate judgments. When students do not gain enough in-depth information to transform it into procedural knowledge during practice, they cannot accurately judge their performances. The limited instructional time dedicated to team sports in physical education may contribute to students’ inaccurate self-assessments. Many school curricula allocate only a few months per year to specific sports, which may not provide students with sufficient exposure to develop expertise in judgment accuracy.

Expanding quality instructional time and offering students the opportunity to frequently estimate their performance accuracy, which serves as immediate feedback [27], gives them the chance to gain the theoretical and experience-based cues they need to enhance self-assessment abilities and their performance quality. Thus, longer instructional periods, combined with targeted activities and student-centered teaching styles such as reciprocal and self-check that promote self-regulated learning [28,29], may help them develop a deeper understanding of performance evaluation.

Overall, the findings of this study highlight the complex interplay between metacognition, knowledge acquisition, and judgment accuracy in motor skill performance. While students demonstrate engagement in self-monitoring and reflection, their ability to accurately judge their performance is limited by gaps in declarative and procedural knowledge. By implementing effective instructional strategies and extending the duration of sports education, educators can support students in developing more accurate self-assessment skills, ultimately enhancing their confidence and competence in physical activities.

### 4.1. Limitations

Although the present study focused on how students justify their performance estimation rather than actual volleyball serve scores, physical characteristics including height, weight, and strength may have hampered their ability to perform as they had planned. Additional limitations were the small sample size and the students’ limited experience in assessing their performance accuracy.

### 4.2. Practical Implications and Future Research

The findings have practical implications for physical educators and coaches, providing insights into how theory- and experience-based elements influence students’ estimation accuracy in sports performance. Based on our research, physical educators and coaches should provide adolescents, particularly females, with more detailed information on their motor skill performances and the chance to regularly evaluate themselves in order to improve their technical and tactical abilities in sports. The availability of safe physical infrastructure and facilities for adolescent girls should also be a top concern for governments, as this would allow females to play sports on an equal basis with males. In this study, the sample size was relatively small. Therefore, to obtain more robust results, future research should examine judgment justifications using a larger sample, across various sports tasks and after repeated judgment trials.

## 5. Conclusions

In many curricula, team sports are taught for only a few months, which may not provide sufficient time for students to develop the theory and experience-based knowledge necessary for accurate self-assessment and confident skill execution. As a result, many students struggle with judgment accuracy and may lack confidence in their abilities.

To enhance students’ ability to accurately assess their performance, physical education programs should extend the duration of instruction, ideally spanning a semester or an entire academic year. Additionally, adopting appropriate teaching styles and incorporating targeted activities and strategies that promote metacognitive awareness and skill refinement can support students in developing high-quality motor skills. A well-structured curriculum that fosters both knowledge and experience-based learning will contribute to students’ long-term engagement in sports and physical activity.

## Figures and Tables

**Table 1:** Questions about students’ justification for judgment prediction in volleyball service.

Questions	Categories
Did you feel confident about your estimation on the first trial? What were your estimation and actual scores?	confidence
When I asked you to perform the second trial, did you keep in mind your first estimation and performance?	monitoring/ reflection
If yes, did you change something in your performance on the second trial? If yes, what was that?	declarative knowledge
How did you perform the second serve? Can you describe and/or perform this?	procedural knowledge
Did the first performance help you? In which way?	experience
Did you believe that the second estimation could be to the actual performance? If you were not sure about your performance, why did you overestimate or underestimate it?	confidence

**Table 2:** Frequencies and percentages for “high” and “low” judgment accuracy students in volleyball service.

	Frequencies	Percentages
“High accuracy” group	13	59.1%
“Low accuracy” group	9	40.9%
Students	22	100%

**Table 3:** Frequencies and percentages for “high” and “low” judgment accuracy for boys and girls in volleyball service.

Gender	Frequencies	High Group	Frequencies	Low Group	Total
Boys	8	61.5%	5	38.5%	13
Girls	1	11.1%	8	88.9%	9

**Table 4:** *t*-test values and descriptive statistics (M, SD) for both groups’ judgment accuracy variables in volleyball service.

Group	“High” Accuracy		“Low” Accuracy		
	M	SD	M	SD	
Confidence 1	1.00	0.000	0.923	0.277	*t*(20) = 0.826 *p* > 0.05
Declarative/procedural	0.777	0.440	0.076	0.277	*t*(20) = 4.59 *p* < 0.001
Experience	1.00	0.000	0.846	0.375	*t*(20) = 1.22 *p* < 0.05
Monitoring	1.00	0.000	0.923	0.277	*t*(20) = 0.826 *p* > 0.05
Confidence 2	0.888	0.333	0.846	0.375	*t*(20) = 0.274 *p* > 0.05

M = mean, SD = Standard Deviation.

**Table 5:** *t*-test values and descriptive statistics (M, SD) for boys’ and girls’ judgment accuracy variables in volleyball service.

Group	“Boys”		“Girls”		
	M	SD	M	SD	
Confidence 1	1.00	0.000	0.888	0.333	*t*(20) = 1.21 *p* < 0.05
Declarative/procedural	0.538	0.518	0.111	0.333	*t*(20) = 2.17 *p* < 0.001
Experience	1.00	0.000	778	441	*t*(20) = 1.83 *p* < 0.001
Monitoring	1.00	0.000	0.888	0.333	*t*(20) = 1.21 *p* < 0.05
Confidence 2	1.00	0.000	0.666	0.500	*t*(20) = 2.43 *p* < 0.001

M = mean, SD = Standard Deviation.

## Data Availability

The data presented in this study are available on request from the corresponding author due to privacy and ethical restrictions.

## Abbreviations

The following abbreviations are used in this manuscript:

AAHPERD	American Alliance for Health, Physical Education, Recreation, and Dance
PE	Physical Education
QPE	Quality Physical Education
